# Correction: Enhanced performance in transparent conducting materials at the interface of a wide band gap semiconductor and a correlated metal

**DOI:** 10.1039/d6mh90087a

**Published:** 2026-08-21

**Authors:** Jessica L. Stoner, Maria Batuk, Troy D. Manning, Matthew S. Dyer, Joke Hadermann, Matthew J. Rosseinsky, Jonathan Alaria

**Affiliations:** a Department of Physics and Stephenson Institute for Renewable Energy, University of Liverpool Oxford Street L69 7ZE UK alariaj@liverpool.ac.uk; b Materials Innovation Factory, Department of Chemistry, University of Liverpool 51 Oxford Street L7 3NY UK m.j.rosseinsky@liverpool.ac.uk; c Electron Microscopy for Materials Science (EMAT), University of Antwerp Groenenborgerlaan, 171 B2020 Antwerp Belgium

## Abstract

Correction for ‘Enhanced performance in transparent conducting materials at the interface of a wide band gap semiconductor and a correlated metal’ by Jessica L. Stoner *et al.*, *Mater. Horiz.*, 2025, **12**, 5820–5828, https://doi.org/10.1039/d5mh00283d.

The authors wish to correct an omission that occurred during the data reduction and analysis of the electrical transport data presented in the published article. Specifically, a standard geometric correction factor of 0.22, required by the sample’s specific measurement geometry, was omitted from the final van der Pauw equations used to calculate the absolute electrical resistivity and sheet resistance.

This omission systematically scaled the absolute magnitudes of the transport data reported in the paper. Consequently, amendments are required to the numerical values of the resistivity, sheet resistance, mobility and Figure of Merit reported in the text and Fig. 3 and 4 of the published article, as detailed in this notice; the corresponding Tables S1 and S2 in the originally published Supplementary Information (SI) file have also been updated accordingly.

In section 2.2 “Electrical Properties”, in the first paragraph, the text “…room temperature resistivity between 40–70 µΩ cm…” should be replaced with “…room temperature resistivity between 200–300 µΩ cm…”; and the text “…between 4.4(8) µΩ cm and 12.8(4) µΩ cm, lower than the lowest reported value…” should be replaced with “…between 19.8(8) µΩ cm and 56.9(4) µΩ cm, lower than the lowest reported value…”. In the second paragraph, the text “…reaching 3.2 × 10^4^ cm^2^ V^−1^ s^−1^ at low temperature compared to 10 cm^2^ V^−1^ s^−1^…” should be replaced with “…reaching 9.5 × 10^3^ cm^2^ V^−1^ s^−1^ at low temperature compared to 2 cm^2^ V^−1^ s^−1^…”.

Fig. 3 should be replaced as shown here.

**Fig. 3:**
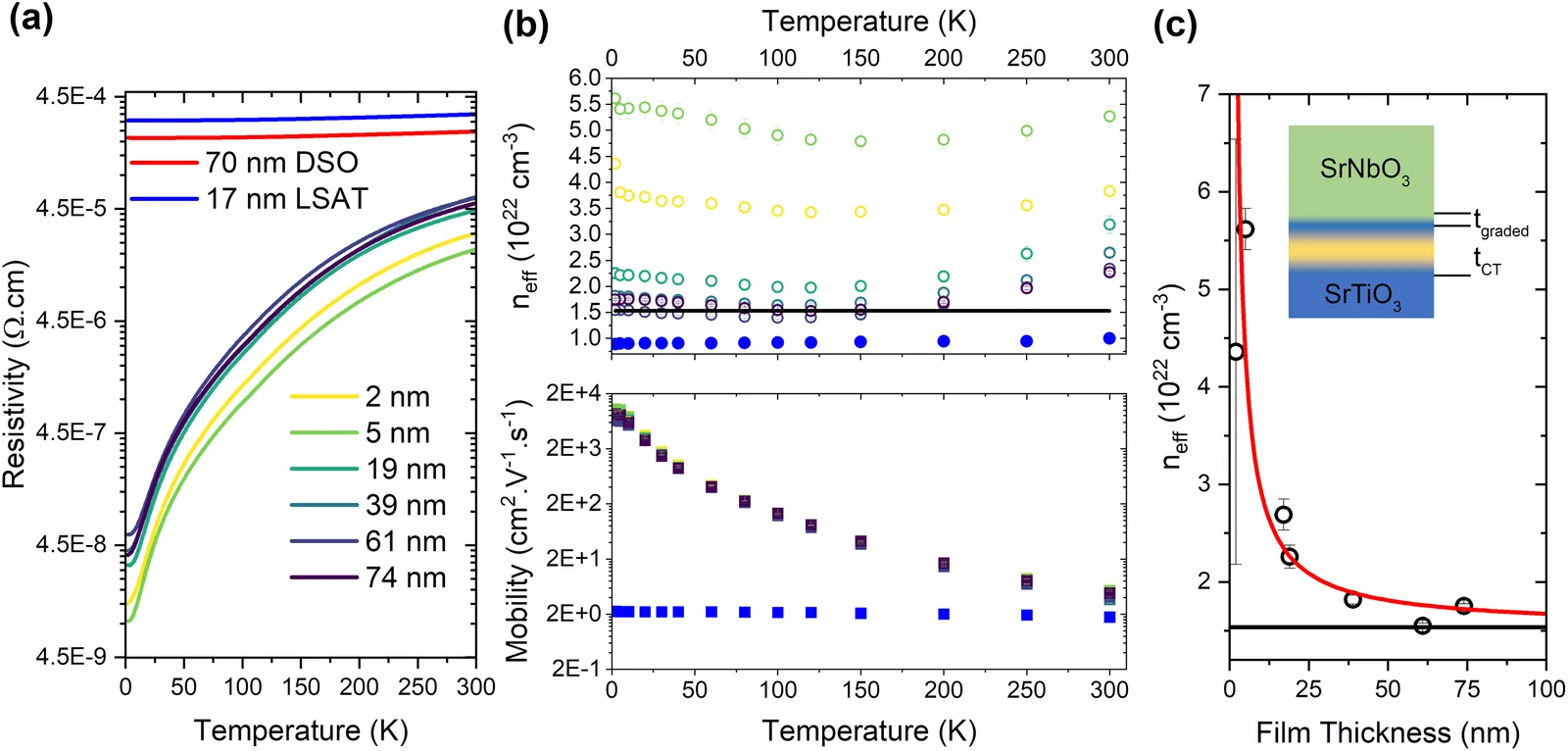
(a) Electrical resistivity as a function of temperature for various thicknesses of SrNbO_3_ heterostructures grown on SrTiO_3_ substrates (yellow to purple line) and thick (red line) and thin (blue line) SrNbO_3_ heterostructures grown on DyScO_3_ and LSAT substrates respectively. (b) Effective carrier concentration extracted from Hall effect (top panel) and associated carrier mobility (bottom panel) as function of temperature. The color scheme is the same as in (a) and the mobilities of all SrNbO_3_ heterostructures grown on SrTiO_3_ substrate overlap. The black line in the top panel represents the theoretical carrier concentration expected for one free electron per unit cell in SrNbO_3_. (c) Effective carrier concentration obtained from Hall effect at 300 K as a function of SrNbO_3_ thickness (black dots). The black line corresponds to the theoretical carrier concentration expected for one free electron per unit cell in SrNbO_3_ and the red line corresponds to the carrier concentration expected with an additional interfacial charge transfer layer of 9 nm as represented in the inset.

In section 2.3 “Improved FOM in the hybrid CM/TCO heterostructure”, at the end of the first paragraph and in Fig. 4 caption, the constant resistivity values 12 µΩ cm (red line in Fig. 4(a), bottom panel) and 5 µΩ cm (blue line in Fig. 4(a), bottom panel), should be replaced with 54 µΩ cm and 23 µΩ cm, respectively. In the second paragraph, the FOM of 0.04 Ω^−1^ should be 0.01 Ω^−1^, and the text “…close to the 0.08 Ω^−1^ record…” should be replaced with “…close to the 0.04 Ω^−1^ record…”.

Fig. 4 should be replaced as shown here.

**Fig. 4:**
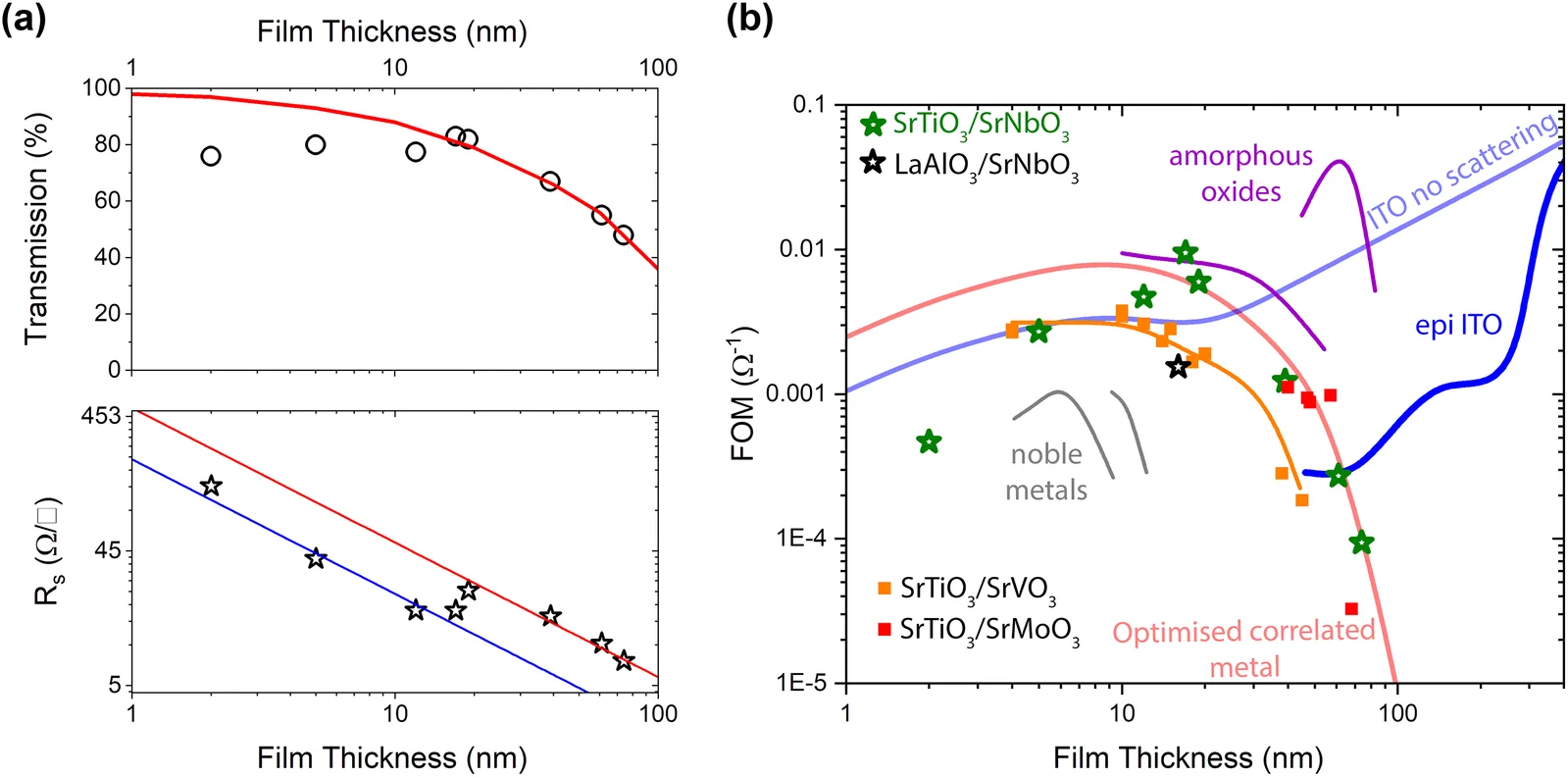
(a) Transmission (black circles top panel) and sheet resistance (empty black stars bottom panel) of SrNbO_3_ heterostructures grown on SrTiO_3_ substrates as function of film thickness. The red line in the top panel is the same transmission calculated for an optimized CM presented in Fig. 1(a). The red and blue lines in the bottom panel correspond to constant resistivities of 12 µΩ cm and 5 µΩ cm respectively. (b) Haacke FOM for the SrTiO_3_/SrNbO_3_ heterostructures as function of thickness (green empty stars) and LaAlO_3_/SrNbO_3_ for the optimum thickness (black empty star). The red and blue opaque line represents the calculated Haacke FOM for an optimized d^2^ CM (red squares are for SrTiO_3_/SrMoO_3_ heterostructure from Stoner *et al.*)^8^ and ITO respectively as presented in Fig. 1(a). Measured Haacke FOM as function of thickness for epitaxial ITO (thick blue line), d^1^ SrVO_3_ CM (orange line and squares), noble metals (grey line) and amorphous oxides (purple line) are reproduced from ref. 28 for comparison.

The authors emphasize that this correction scales only the magnitudes of the transport properties by a uniform constant. It does not modify the observed temperature dependencies, or qualitative conclusions detailed in the original manuscript. The overarching physical interpretation and scientific claims of the paper remain entirely unaffected and valid.

The Royal Society of Chemistry apologises for these errors and any consequent inconvenience to authors and readers.

